# Application of Deep Learning on the Prognosis of Cutaneous Melanoma Based on Full Scan Pathology Images

**DOI:** 10.1155/2022/4864485

**Published:** 2022-08-28

**Authors:** Anhai Li, Xiaoyuan Li, Wenwen Li, Xiaoqian Yu, Mengmeng Qi, Ding Li

**Affiliations:** ^1^Department of Dermatology, Qingdao Huangdao District Central Hospital, Qingdao, Shandong, China; ^2^Department of Traditional Chinese Medicine, The Affiliated Hospital of Qingdao University, Qingdao, Shandong, China; ^3^Department of Hematology, Qingdao Women and Children's Hospital, Qingdao, Shandong, China; ^4^Department of Dermatology, Qingdao Haici Hospital (Qingdao Traditional Chinese Medicine Hospital), Qingdao, Shandong, China; ^5^Department of Endocrinology, The Affiliated Hospital of Qingdao University, Qingdao, Shandong, China

## Abstract

**Introduction:**

The purpose of this study is to use deep learning and machine learning to learn and classify patients with cutaneous melanoma with different prognoses and to explore the application value of deep learning in the prognosis of cutaneous melanoma patients.

**Methods:**

In deep learning, VGG-19 is selected as the network architecture and learning model for learning and classification. In machine learning, deep features are extracted through the VGG-19 network architecture, and the support vector machine (SVM) model is selected for learning and classification. Compare and explore the application value of deep learning and machine learning in predicting the prognosis of patients with cutaneous melanoma.

**Result:**

According to receiver operating characteristic (ROC) curves and area under the curve (AUC), the average accuracy of deep learning is higher than that of machine learning, and even the lowest accuracy is better than that of machine learning.

**Conclusion:**

As the number of learning increases, the accuracy of machine learning and deep learning will increase, but in the same number of cutaneous melanoma patient pathology maps, the accuracy of deep learning will be higher. This study provides new ideas and theories for computational pathology in predicting the prognosis of patients with cutaneous melanoma.

## 1. Introduction

Cutaneous melanoma (CM) is the most dangerous type of skin cancer; it is the fifth most common cancer in men and women in the United States [1]. The incidence of CM increases with age [2]. It is estimated that there will be 106110 new patients in 2021 and result in 7180 deaths [3]. Thanks to the application of immunotherapy and targeted therapy drugs, the survival rate of CM patients has been continuously improved [4]. But it still cannot change the poor prognosis of patients with advanced CM [5]. Therefore, the early diagnosis of CM is essential to improve the survival rate of CM patients and save lives, because the survival rate of CM patients depends on the stage of the disease at the time of diagnosis [6].

In recent years, bioinformatics analysis methods based on whole genome sequencing have made great progress in the discovery of CM diagnosis markers, prognosis prediction, CM subtypes classification, and personalized treatment development [7, 8]. However, the expensive cost of whole genome sequencing brings a greater economic and social burden and cannot be widely cited [9]. With the deepening of deep learning research in the field of medicine. New deep learning network structure methods continue to emerge, deep learning can choose different neural network architectures to study and classify different types of medical examination results, including whole genome sequencing result, medical imaging data, pathological picture, common laboratory indicators and patient written electronic health record [10–13]. The research of Rahaman et al. and Chen et al. also proved the great value of deep learning in clinical direction, and with the deepening of algorithm research, the application value of deep learning in medical treatment is limitless [14–17]. Among them, the VGG-19 network structure was found to have high accuracy in applications for diagnosing retinal diseases and identifying thyroid tumor types [18, 19]. Thus, the development of a new analysis method based on pathological images to predict the prognosis of CM patients based on deep learning is of great significance to the diagnosis and prognosis of CM patients.

This research is based on the CM patient cancer tissue pathology full scan from the TCIA database through deep learning method study and detects whether deep learning can effectively distinguish patients with different prognoses. This research is aimed at discussing the apply value of deep learning in the prediction of CM patient prognosis and offering a new and cheap detection method for the clinical dermatologist.

## 2. Materials and Method

### 2.1. Data Collection and Preprocessing

The cutaneous melanoma pathology images (ID: CPTAC-CM) [20] were downloaded from The Cancer Imaging Archive (TCIA) database (https://www.cancerimagingarchive.net/) [21], including a total of 12 deceased cutaneous melanoma patients (51 melanoma pathological pictures) and 59 living cutaneous melanoma patients (272 melanoma pathological pictures), and divided into deceased cutaneous melanoma patients group (DCM) and living cutaneous melanoma patients group (LCM). In order to better perform deep learning of the pathology map, the svs format of the pathology map is converted to the png format through the reaConverter 7 pro software (https://www.reaconverter.com/).

### 2.2. VGG-19 Model Construction

In this section, we choose the VGG-19 model as the network architecture and learning model. The VGG-19 model was constructed using Python 3.6 (https://www.python.org/). The patients in our research cohort are also completely randomly assigned. 80% of the total sample is set as the training set, 20% is set as the validation set, and 20% of the training set is set as the test set. This binary classification mode, batch size was set as 2048, epoch was set as 200, weight was chosen as ImageNet model, and activation function was set as sigmoid. The optimizer was selected as Adam (learning rate = 0.01, beta_1 = 0.9, beta_2 = 0.999, epsilon = 1*e* − 08, decay = 0.0). The 5-fold cross-validation was used to evaluate the accuracy of this model.

### 2.3. Deep Feature Extraction and SVM Model Construction

In this section, we choose VGG-19 model as the network architecture to extract these pathology scan deep features and to quantify these pathology maps for follow-up research on machine learning; weight was chosen as ImageNet model. In order to better perform subsequent machine learning analysis, filter the acquired depth features before importing them into lasso regression analysis to, and delete the blank values and unqualified depth features. Finally, a support vector machine (SVM) model is established based on the optimal depth features selected, and the prediction model is verified in the test set.

### 2.4. Statistical Analysis

Statistical analysis was performed using Python 3.6 and GraphPad Prism 9.0 software (https://www.graphpad.com/; San Diego, United States). Using the prognosis results as the gold standard, we calculated the sensitivity and specificity of the machine learning model and deep learning model, plotted the receiver operating characteristic (ROC) curve, and calculated the area under the ROC curve (AUC), thus evaluating the prediction performance of the model.

The chi-square test or Fisher's exact test was used to compare the differences in count data between the two groups, and the independent sample *t*-test or a nonparametric test was used to compare the differences in measurement data. A value of *P* < 0.05 was considered statistically significant.

## 3. Result

### 3.1. CM Patient Detail

There are a total of 53 CM patients after deleting the patients who did not indicate the survival status, including 12 deceased CM patients (51 melanoma pathological pictures) and 41 living CM patients (271 melanoma pathological pictures) (Table 1).

### 3.2. Diagnostic Performance of Deep Learning Model

**Figure 1:**
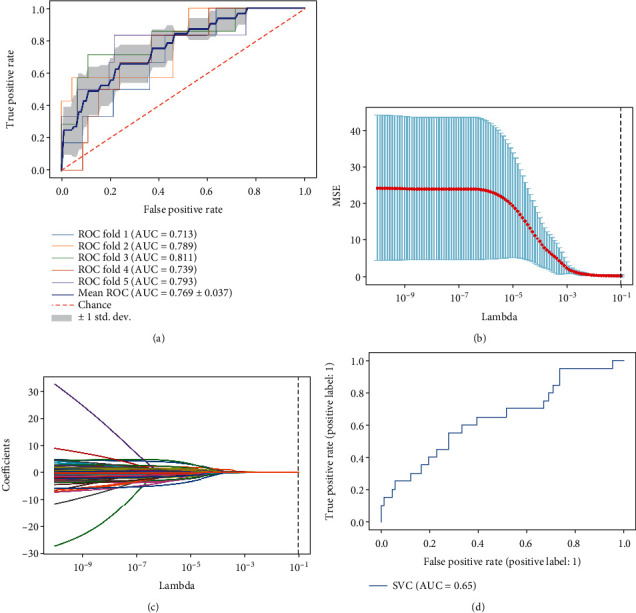
(a) Receiver operating characteristic (ROC) curves and area under the curve (AUC) of the nomogram for the accuracy of deep learning model diagnosis. (b, c) Plots for lasso regression coefficients over different values of the deep feature from pathology scan images. (d) Receiver operating characteristic (ROC) curves and area under the curve (AUC) of the nomogram for the accuracy of machine learning model diagnosis in the testing set.

In this section, we through VGG-19 network architecture directly learn and classify CM patients with good prognosis and CM patients with poor prognosis. The result of the 5-fold cross-validation showed the ROC fold 1 (AUC = 0.713), the ROC fold 2 (AUC = 0.789), the ROC fold 3 (AUC = 0.811), the ROC fold 4 (AUC = 0.739), the ROC fold 5 (AUC = 0.793), and the mean ROC of 5 ROC fold (AUC = 0.769 ± 0.037). The results suggest that the best AUC for 5-fold cross-validation is fold 3 (Figure 1(a) and Tables 2 and 3).

### 3.3. Diagnostic Performance of Machine Learning Model

In this section, we first extract a total of 512 deep features from the pathological pictures of CM patients through the VGG-19 neural network and imported 512 deep features into lasso regression analysis for screening. Finally, we build the SVM model through the screened depth features, and the result showed that the AUC in the validation set is 0.65 (Figures 1(b)–1(d)).

## 4. Discussion

CM is one of the most dangerous types of skin cancer and the main leading cause of cancer-related mortality due its metastatic power and late diagnosis [22]. Thus, predicting the prognosis of melanoma patients in advance is essential to identify CM patients with potentially poor prognoses [23]. How to predict the prognosis of CM patients in advance has also become one of the research hotspots of CM. Bioinformatics analysis methods, metabolomics and intestinal flora, and other methods have proposed methods for predicting the prognosis of CM patients [24–26], which have played an important role in the prognosis research of CM. The application of CNN in clinical medicine provides a new direction and new dimension for the prognosis prediction of CM patients. At present, CNN-related research is mainly based on learning and predicting the skin surface of CM patients, such as the research of Yang et al. and Haenssle et al. [27, 28]. There are only a few studies and reviews on pathological pictures, such as Wang et al.'s article and Laury et al.'s article [13, 29]. There are very few studies based on CNN to predict the prognosis of CM patients by learning from pathological scans.

The histopathological analysis is one of the main gold standards of CM diagnosis, and there are numerous histologic criteria used to diagnose melanoma, but none alone is sufficient to establish CM diagnosis [30]. In order to improve the accuracy of CM diagnosis based on histopathological, multiple researchers offer multiple methods such as immunohistochemical and immunofluorescence [31, 32]. The application of immunohistochemistry and immunofluorescence has improved the accuracy of CM diagnosis to a limited extent, but at the same time, it has also brought an excessively high economic burden. The appearance of deep learning in the medical maybe a better way to solve above questions. Deep learning is a computer model that extracts information from images on a computer. It is possible to extract special data from medical images that are invisible to the human eye and can be used to inform molecular status, prognosis, and treatment sensitivity [33, 34]. Related research has found that deep learning can not only provide the accuracy and objectivity of diagnosis but also reduce the workload and inspection cost of pathologists, because deep learning can learn pathology full scan pictures without the pathologist providing any part of the comment area [35, 36]. It is precise because the integration of deep learning and histopathology has promoted the rapid development of computational pathology. In addition to the routine diagnosis of histopathology, computational pathology also has the ability to identify and extract new features of diseases [37]. Related research results show that computational pathology can effectively predict lymph node metastasis detection and improve breast cancer Ki67 score, prostate cancer Gleason score, and melanoma tumor infiltrating lymphocyte score [38–40].

In this study, we through machine learning and deep learning constructed a two-classification model to study and classify the pathological scan images separately and to compare the application value of two learning methods on the pathological scan images. The accuracy of the two models showed that the accuracy of the CNN model (AUC = 0.769) is significantly higher than that of the SVM model (AUC = 0.65). We initially thought that compared with the SVM model algorithm, the CNN model algorithm is more suitable for predicting the prognosis of CM patients.

This study is based on CNN learning and classifying pathological scan images of CM patients with different prognoses to evaluate the prognostic value of CNN in predicting CM patients. Compared with the conventional prognostic prediction methods at this stage, CNN can let clinicians know the prognosis of CM patients earlier so that targeted treatment of CM patients can be carried out earlier to improve the prognosis of CM patients. Although in this study, the CNN accuracy ratio did not reach a very satisfactory 90% or more, we believe that as the number of subsequent pathological images increases, the accuracy will increase with the increase in the amount of learning.

## Figures and Tables

**Table 1:** Clinical characteristics of the CM patients.

Parameter	Classification	Individuals	Living	Deceased
Gender	Male (1)	29	23	6
	Female (0)	24	18	6
Age	<60	19	16	3
	≥60-80≤	28	21	7
	>80	5	3	2
BMI	<18.5	0	0	0
	≥18.5-25≤	18	11	7
	>25-30≤	19	17	2
	>30	16	13	3
Stage	Stage I	7	7	0
	Stage II	29	23	6
	Stage III	14	8	6
	Stage IV	3	3	0

**Table 2:** The result of 5-fold cross-validation.

Fold	Classification	Precision	Recall	F1-score	Support
Fold 1 valid	Living	0.838	0.886	0.861	35
	Deceased	0.000	0.000	0.000	6
Fold 2 valid	Living	0.892	0.943	0.917	35
	Deceased	0.500	0.333	0.400	6
Fold 3 valid	Living	0.889	0.914	0.901	35
	Deceased	0.400	0.333	0.364	6
Fold 4 valid	Living	0.838	0.912	0.873	34
	Deceased	0.250	0.143	0.182	7
Fold 5 valid	Living	0.846	0.971	0.904	34
	Deceased	0.500	0.143	0.222	7

**Table 3:** The accuracy of each fold cross-validation.

Fold	F1-score accuracy	Support
Fold 1 valid	0.756	41
Fold 2 valid	0.854	41
Fold 3 valid	0.829	41
Fold 4 valid	0.780	41
Fold 5 valid	0.829	41

## Data Availability

The data used in this study come from the CPTAC-CM dataset of the TCIA database (https://www.cancerimagingarchive.net/).
